# Microplastic-Induced Oxidative Stress in Metolachlor-Degrading Filamentous Fungus *Trichoderma harzianum*

**DOI:** 10.3390/ijms232112978

**Published:** 2022-10-26

**Authors:** Anna Jasińska, Sylwia Różalska, Volha Rusetskaya, Mirosława Słaba, Przemysław Bernat

**Affiliations:** Department of Industrial Microbiology and Biotechnology, Faculty of Biology and Environmental Protection, University of Lodz, Banacha Street 12/16, 90-237 Lodz, Poland

**Keywords:** microplastic, *Trichoderma*, metolachlor, biodegradation, oxidative stress, phospholipids

## Abstract

While there has been intensive research on the influence of microplastics (MPs) on aquatic organisms and humans, their effect on microorganisms is relatively little-known. The present study describes the response of the *Trichoderma harzianum* strain to low-density polyethylene (LDPE) microparticles. MPs, either separately or with metolachlor (MET), were added to the cultures. Initially, MP was not found to have a negative effect on fungal growth and MET degradation. After 72 h of cultivation, the content of fungal biomass in samples with MPs was almost three times higher than that in the cultures without MPs. Additionally, a 75% degradation of the initial MET was observed. However, due to the qualitative and quantitative changes in individual classes of phospholipids, cell membrane permeability was increased. Additionally, MPs induced the overproduction of reactive oxygen species. The activity of superoxide dismutase and catalase was also increased in response to MPs. Despite these defense mechanisms, there was enhanced lipid peroxidation in the cultures containing the LDPE microparticles. The results of the study may fill the knowledge gap on the influence of MPs on filamentous fungi. The findings will be helpful in future research on the biodegradation of contaminants coexisting with MPs in soil.

## 1. Introduction

Plastic particles of varying shapes (such as granules, spheres, threads, fibers, and powder) and sizes (ranging from 1 μm to 5 mm) are called microplastics (MPs). Primary MPs are produced synthetically and used in personal care products, such as toothpaste, shampoo, shower gel, and washing powders. Secondary MPs are formed from larger pieces of plastic during various mechanical, chemical, and biological processes, such as erosion, photooxidation, and corrosion. Their main sources are the abrasions on car tires, road markings, city dust, and synthetic clothes [1,2,3,4,5]. Plastic packaging waste, such as plastic films, bags, food packaging, and other disposable plastics, is also an extensive source of MPs. For example, plastic bags are easily photodegraded, which results in the formation of microparticles [6,7,8]. MPs are present in the marine environment, freshwater habitats, terrestrial ecosystems, and the atmosphere. Several populated areas, such as suburban soil or sediments in a seaport environment [9,10], and areas with sparse human impact and extreme environments, such as glaciers, caves, mountains, regions of Antarctica, and forests [11,12,13,14,15], have traces of contamination by MPs. According to Law et al., in the years 1972–1985 and 2002–2012, there was an increase of about ten times in the average amount of MPs on the surface layer of the North Pacific gyrus [16]. Large masses of micro- and macroplastics are accumulated, creating garbage patches in the gyres of the North Atlantic, South Atlantic, North and South Pacific, and Indian Oceans. The largest garbage patch—called the Great Pacific Garbage Patch—which has an area of 1.6 million km², contains 45–129 thousand tons of plastic [17]. There is a high content of MPs found in coastal waters, coasts, beaches, and sediments from harbors [18,19]. Urban-Malinga et al. examined 12 beaches on the Polish coast (southern Baltic) and found that the average MP concentrations ranged from 76 to 295 elements per kg dry sediments [20]. Some reviews have shown the presence of MPs on surface and groundwater, while only limited studies have been conducted on the content of MPs in terrestrial systems, where significant amounts of MPs are accumulated in soil due to the use of sewage sludge as fertilizer [21,22,23,24]. Plastic mulching and littering are other sources of MPs in soil [25,26]. Such practices are common in agricultural fields and can result in MPs being absorbed by plants and transferred along the food chain.

Several studies on the content of MPs in aquatic organisms revealed traces of MPs in the edible tissues of marine fish, molluscs, and crustaceans [27]. Recent reports mention the presence of MPs in human blood, feces, lungs, and placenta [28,29,30,31]. MPs may harm humans via both physical and chemical pathways. For example, MPs can be physically retained in the digestive tract. On the other hand, chemical transformations may lead to the leaching of plastic additives into the tissues. Research has demonstrated an enhanced inflammatory response, size-related toxicity of plastic particles, and disruption of the gut microbiome. Its harmfulness results from the biopersistent character of MPs and their exceptional hydrophobicity and surface chemistry. Furthermore, chemical additives that improve the properties of plastic and pollutants adsorbed on the surface of the plastic may cause toxic effects including reproductive toxicity (e.g., bisphenol A), carcinogenicity (e.g., vinyl chloride), and mutagenicity (e.g., benzene) [32].

The influence of MPs on aquatic organisms and humans is a topic of constant interest and is becoming better understood. However, the effects of MPs on microorganisms are relatively unclear. Most of the available information concerns the impact of MPs on microbial communities in both aquatic and terrestrial environments, the colonization of MP particles by microorganisms, and the possible biodegradation of polymers [33].

Recently, several reports have shown the direct effect of plastic microparticles on bacteria and microalgae resulting in a change in the growth rate, oxidative stress, and the disturbances in the structure and function of cells. Sun et al. exposed the marine bacterium *Halomonas alkaliphila* on polystyrene nano- and microparticles [34]. The authors observed decreased cell viability and enhanced extracellular polymer production. The *H. alkaliphila* cells reacted to the toxic effects of MPs through increased intracellular reactive oxygen species (ROS) generation.

The present work may be the first study on the influence of low-density polyethylene (LDPE) microparticles on the soil-borne fungus *Trichoderma harzianum* KKP 534. In our previous study, the fungus was found to promote plant growth and limit the development of pathogenic fungi, such as *Fusarium*, *Pythium*, and others [35]. The present study assessed the effect of MPs on the growth of the fungus and the degradation of metolachlor (MET), which is a chloroacetanilide herbicide commonly used in cereal crops. Additionally, the study assessed the change in the phospholipid composition of the cell membrane and its permeability. In order to indicate the level of oxidative stress, the content of ROS and activity of antioxidative enzymes was measured. For the first time, this work indicates the influence of LDPE microparticles on the biodegradation potential of fungi. It also proves that the presence of MPs results in oxidative stress induction. More research in this area is necessary considering that the MPs and pesticides coexist in soils (especially agricultural ones).

## 2. Results and Discussion

### 2.1. Does MP Influence the Growth of T. harzianum and the Degradation of MET?

**Figure 1:**
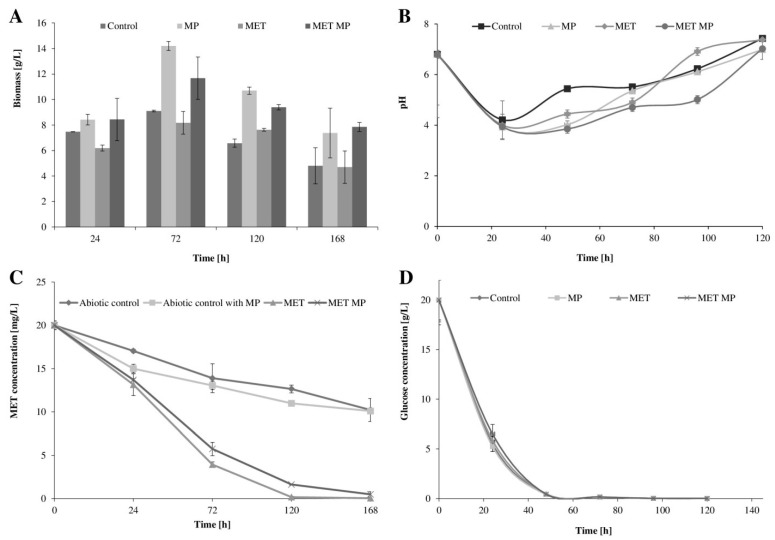
Fungal biomass production (**A**), culture pH value (**B**), MET concentration (**C**), and glucose consumption (**D**) during *T. harzianum* cultivation with MPs (2 g L^−1^), MET (50 mg L^−1^), both, or without pollutants. MP—culture with microplastics; MET—culture with metolachlor; MET MP—culture with both metolachlor and microplastics.

Our previous study indicated that *T. harzianum* KKP 534 exhibited high resistance to MET present in the growth environment [36,37]. Additionally, the microorganism can eliminate compounds such as MET from the growth medium. So far, however, the influence of MPs on the growth of *T. harzianum* and its biodegradability remains unknown. In the present study, LDPE microparticles (2 g L^−1^) were added to the growth medium, either separately or along with MET (at 50 mg L^−1^ concentration). There was a significant improvement in fungal growth with the addition of LDPE microparticles (Figure 1A). The differences were most noticeable during the stationary phase of the growth. The content of dry mass (g L^−1^)—determined after 72 h in culture—containing MPs was almost three times higher than that in the control culture without MPs. Several studies have described the inhibitory effect of polyethylene (PE) microparticles on microorganisms. According to Ustabasi and Baysal, PE extracted from toothpaste had an inhibitory effect on four bacterial strains isolated from seawater [38]. The authors observed a reduction in bacterial growth, changes in protein metabolism, and/or an increase in lipid peroxidation. A similar effect was observed in *H. alkaliphila* bacteria cultured in the presence of polystyrene nano- and microparticles [34]. According to Pramila and Ramesh, the hydrophobic LDPE film can act as a substrate for some groups of microorganisms which can form a biofilm on it [39]. Additionally, powdered LDPE added to minimal medium was utilized as a carbon source even without a nitrogen source. According to these data, the use of plastic microparticles as an additional source of carbon and energy may result in increased production of biomass. Further, the presence of MPs slightly alleviated the negative effect of MET on the growth of *T. harzianum*. The production of biomass in the cultures containing MET was found to be about 10–17% lower than that in the control cultures. However, with the addition of MPs, the biomass content reached close to that of the control system or even higher, and this could be the result of MET adsorption on the MP surface. Simultaneously, there was a decrease in pH after the first 24–48 h of cultivation (Figure 1B). Among the tested samples, those containing both MPs and MET had the lowest pH values. This suggests that *Trichoderma* spp. grow better in acidic conditions, with optimal growth at pH ranging from 4 to 6, and can modify the pH value of the environment [40].

The LC–MS technique was used to assess the MET concentration in the cultures, and the determined values were compared to the concentration in abiotic controls (Figure 1C). The rate of elimination of MET after 24 h of cultivation was about 25%. However, extending the incubation time to 72 h contributed to removing over 75% of pesticide in the systems containing MET and in those containing MET with MPs. These results are consistent with those of previous studies, which indicate the acclimatization of the microorganism as the first stage preceding the efficient elimination of the toxic substance from the growth environment [41]. This effect was accompanied by balanced consumption of glucose in the medium (Figure 1D). To the best of our knowledge, this is the first study to assess the effect of MPs on the microbial biodegradation of MET. However, the topic requires immediate attention due to the continuous coexistence pesticides and microparticles of plastic in the environment (especially in soil).

### 2.2. Does MPs Disturb T. harzianum Cell Membranes?

In the presence of toxic compounds or other unfavorable environments, the cell membrane is the first line of defense of the microbial cell. Microorganisms can modify their cell membrane in response to stress factors, mainly by changing the composition of membrane lipids or by modifying the length, branching, and saturation of acyl chains of fatty acids constituting membrane lipids. The modifications can change the membrane surface charge, fluidity, curvature, and thickness. Lipidomic studies focus mainly on phospholipid analysis to assess the modifications taking place in the cell under the influence of stress factors. The main membrane phospholipids are phosphatidylcholine (PC), phosphatidylethanolamine (PE), phosphatidylserine (PS), phosphatidylinositol (PI), and phosphatidic acid (PA).

#### 2.2.1. Phospholipids Composition

The present study analyzed the changes in membrane phospholipids under the influence of MPs added separately or along with MET. As seen in Table 1, the major classes of phospholipids identified in *T. harzianum* cultures were PC and PE. This is in line with previous data on *Trichoderma* fungi [36,42,43]. The content of PC and PE were in the ranges of 15.50–66.44 and 29.85–73.84% of total cell phospholipids, respectively. The PC content reduced significantly with the addition of MPs to the medium. However, the PE level in the total phospholipids pool was increased. The changes in the cultures containing MPs were most noticeable in the first 48 h of cultivation. The PC content in the mycelium in the 24 h control cultures was 44.32% (compared to total phospholipids); however, the PC contents in the biomass grown in the presence of MPs or both MPs and MET decreased to 27.24 and 29.46%, respectively. Interestingly, there were no significant differences in PC content in the mycelium obtained from the cultures containing only MET. Concurrently, it was found that as the PC content decreased, the PE level increased in the mycelium from cultures grown in the presence of MPs or both MPs and MET. The PE level after 24 h of cultivation achieved 62.46 and 63.18%, respectively. The PE content in the control system was 48.33%. The changes in the content of PE and PC influenced the PC/PE ratio. This parameter gives information about the integrity of the cell membrane. An increase in the PC/PE ratio stabilizes the lipid bilayer, while its decrease may increase membrane permeability, which in turn may disturb the integrity of the cell [44].

Additionally, there was a significant increase in the level of PI. The content of this phospholipid in the control system was 3.63%, while the content in the cultures with MPs, MET, and a mixture of both pollutants more than doubled. Furthermore, a slight increase in the content of PS and PA was noted as a result of the presence of contaminants. These changes persisted in the mycelium grown for 48 h. However, after 72 h of cultivation, the content of all the analyzed classes of PLs was similar in the mycelium from cultures grown in the presence of contaminants and in their absence (control). This correlates with the above results which indicate enhanced elimination of MET after 72 h of cultivation.

Based on the presented findings, it can be concluded that the presence of plastic microparticles in the growth environment has a significant impact on the *T. harzianum* cells. This study is the first work describing the disturbance of fungal cell membranes due to MPs. The response of *T. harzianum* cells to the plastic microparticles as a stress factor seems to be destabilization of the membrane and an increase in its permeability. As described previously, during the action of organic tin compounds, pesticides, and heavy metals, fungi of the genus *Metharizium*, *Cuninghamella*, and *Paecilomyces* exhibit a similar mechanism of adaptation [45,46,47,48,49].

#### 2.2.2. Fatty Acids

Due to unfavorable environmental factors, the abundance of phospholipids, and the length and saturation degree of acyl chains that form fatty acids can change. Thus, the study examined the total content of fatty acids and the major phospholipids species. The major cellular fatty acids in fungi are palmitic (16:0), stearic (18:0), oleic (18:1), and linoleic (18:2) acid [50]. As shown in Table 2, palmitic acid (31.89–37.70% of total cell fatty acids) was among the most abundant compounds in *T. harzianum* mycelium (obtained from either control cultures or cultures with MET, MPs, or both). Due to the addition of MPs and/or MET to the culture, there was a decrease in palmitic and stearic acid, with a simultaneous increase in the proportion of oleic and linoleic acids. Therefore, the value of the unsaturation index (UI) changed. The value of UI was 66.10 for the control system, while it was 80.06, 83.57, and 75.47, respectively, for the mycelium grown in the presence of MPs, MET, or a mixture of these contaminants. However, the degree of unsaturation of the fatty acids was highest in the systems containing MPs or MET, both added separately. This confirms the previous findings indicating that MPs could mitigate the toxic effects of MET on fungal cells.

**Figure 2:**
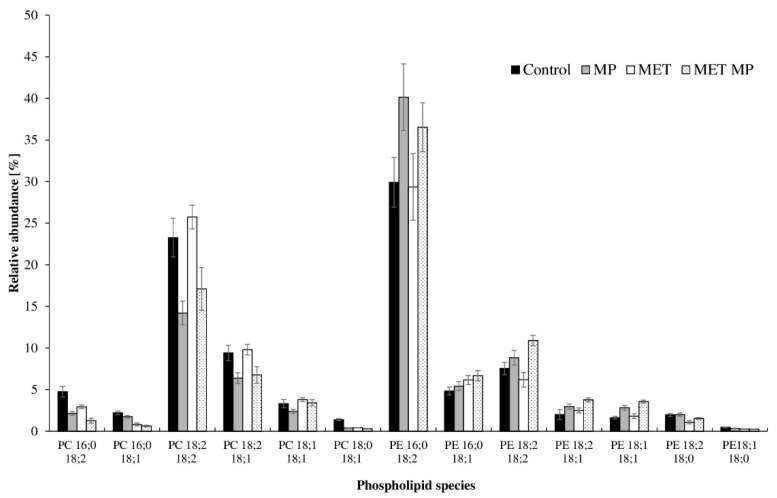
The variation of the PC and PE molecular species determined in the *T. harzianum* cells after 24 h of cultivation with MPs (2 g L^−1^), MET (50 mg L^−1^), MPs and MET mixture, or without the tested pollutants (control). MP—culture with microplastics; MET—culture with metolachlor; MET MP—culture with both metolachlor and microplastics.

The LC–MS/MS analysis revealed that PC 18:2 18:2, PC 18:2 18:1, and PE 16:0 18:2, PE 18:2 18:2 are the major molecular species of phospholipids in *T. harzianum* cells (Figure 2 and Table S1). MPs (either alone or with MET) noticeably decreased the level of unsaturated PC 18:2 18:2 from 23.27% (control) to 14.21% (MPs) and 17.10% (MPs with MET). Additionally, due to the presence of MPs in the growth medium, there was a decrease of PC 18:2 18:1 with a simultaneous increase of PE 16:0 18:2 and PE 18:2 18:2. Interestingly, all the changes were only detected in the first 24 h of cultivation. Other significant differences between the control and the MP-containing cultures were observed after 72 h.

Modifications of individual fatty acid fractions and the amount of polyunsaturated fatty acids are important for the response of microorganisms to stress caused by unfavorable environmental factors, including pollutants [51]. An increase in the level of linoleic acid (C 18:2) was reported for *Umbelopsis isabelina* and *Cuninghamella elegans* incubated with 2,4-D and quinoline, respectively [52,53,54]. Similarly, due to linoleic acid enrichment of cultures, a significant rise in phospholipid unsaturation was noted in *Paecilomyces marquandii* cultures incubated with Zn ions [46]. Previously, You et al. studied the *Synechocystis* sp. cell membrane rearrangement resulting from the influence of ciprofloxacin and polystyrene nano- or microparticles [55]. Additionally, Qin et al. observed increased biosynthesis of unsaturated fatty acids by zebrafish larvae during their exposure to polystyrene nanoparticles [56]. However, this is the first report on changes occurring in fungal membrane lipids during exposure to PE microparticles.

#### 2.2.3. Membrane Permeability

With reference to the previously obtained results, the propidium iodide fluorescence assay was used to assess the condition of the fungal membrane exposed to MPs. Propidium iodide is a dye that passes through the disrupted cell membrane, though it is impermeable to intact cells. The assay results suggested an increase in the membrane permeability of *T. harzianum* hyphae after 24 h of incubation with MPs and/or MET (Table 3). For samples cultivated with MPs, MET, and both pollutants, the fluorescence intensity after biomass incubation with propidium iodide reached 51.847, 45.799, and 20.119 U mg^−1^, respectively. For the control samples (cultivated without MET and MPs), the fluorescence intensity was 18.231 U mg^−1^ of dry biomass. However, there was no increase in permeability after 72 or 120 h of cultivation.

Micro- and nanoplastics can damage cell membranes. According to researchers, through this mechanism, MPs can increase gene transfer between bacteria, thus inducing antibiotic resistance [57]. The membrane damage caused by MP particles may result in the disruption of cell integrity, which has been observed in bacteria and algae. Yan et al. found that increasing the concentration of polystyrene micro- and nanoparticles from 50 to 500 mgL^−1^ significantly increased the membrane permeability of *Chlamydomonas reinhardii* cells [58]. Additionally, Liu et al. observed that the size- and concentration-dependent damage of *Streptomyces coelicolor* cells resulted in enhanced membrane permeability [59]. They concluded that cell damage and change in membrane permeability may arise due to oxidative stress. However, currently, the effects of MPs on the integrity of fungal cells are unknown.

### 2.3. Does MPs Induce Oxidative Stress in T. harzianum Cells?

Many studies proposed oxidative stress as a major mechanism of MP-induced toxicity. Previously, MP-induced oxidative stress was detected in numerous aquatic organisms, such as *Mytilus edulis* [60], *Artemia franciscana* [61], *Eriocheir sinensis* [62], and *Dicentrarchus labrax* [63].

#### 2.3.1. ROS Level

The mechanisms of MP toxicity could be associated with the increased production of intracellular ROS. The primary mechanism of polystyrene nanoparticles was considered to be the adsorption of these nanoparticles onto the *Escherichia coli* cell surface and the subsequent increase of intracellular ROS [64]. Additionally, Sun et al. studied the toxicity of polystyrene to the marine bacterium *H. alkaliphila* [34]. The results indicated that polystyrene induced oxidative stress and inhibited the growth of the bacteria. Therefore, in the present study, the intracellular level of ROS in *T. harzianum* cells exposed to the MPs and/or MET was determined. The H_2_DCFDA assay was used as a nonspecific indicator [65].

**Figure 3:**
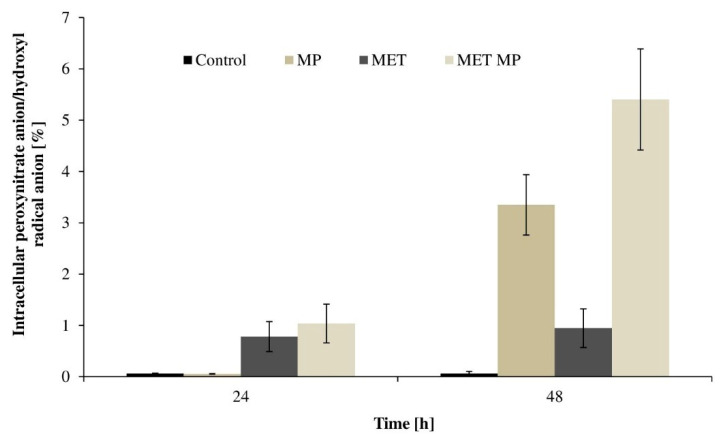
ROS generation (HO•/ONOO^−^) in *T. harzianum* cells after 24 and 48 h exposure to MPs (2 g L^−1^), MET (50 mg L^−1^), MPs and MET mixture, or without the tested pollutants (control). MP—culture with microplastics; MET—culture with metolachlor; MET MP—culture with both metolachlor and microplastics.

The results presented in Figure 3 indicate that the LDPE microparticles induced ROS production by *T. harzianum*. In the mycelium obtained from the 24 h cultures, the level of ROS was found to be increased only in the cells grown with MET and with both MPs and MET. The ROS content determined in these cells was 0.781 and 1.037%, respectively, while in cells from the control system, it was only 0.029%. However, in the mycelium obtained from the 48 h mycelium, the ROS level was more than 56 and 91 times higher in the cells from the cultures containing MPs and both MPs and MET, respectively, compared to that in the control system. These results correspond to the data on the condition of the *T. harzianum* cell membranes during MP exposure, which indicates that the membranes may probably become unsealed and damaged due to the action of MP. This suggests that MPs and MET may generate intracellular perturbations which result in the development of oxidative stress. This is the first report of MP-induced oxidative stress in fungi.

#### 2.3.2. Antioxidative Enzymes Activity

Enzymatic as well as non-enzymatic antioxidative agents, such as superoxide dismutase (SOD), catalase (CAT), ascorbate peroxidase, ascorbic acid, glutathione, and phenolic compounds, counteract the toxic effects of ROS [66]. Han et al. studied the effects of micro- and nanoplastics on the freshwater crayfish, *Procambarus clarkii* [67]. The authors observed an increase in the activity of antioxidant agents, such as glutathione, SOD, acid phosphatase, lysozyme, and glutathione peroxidase. Additionally, there was a reduction in the number of probiotic bacteria colonizing the crayfish intestinal tract, which might suggest the adjunctive role of *Lactobacillus* in the treatment of oxidative stress in *P. clarkii* cells exposed to polystyrene nanoparticles. Among enzymatic antioxidants, the most effective in filamentous fungi is SOD, which catalyzes the conversion of toxic superoxide anions to relatively less harmful H_2_O_2_, and CAT, which converts H_2_O_2_ to H_2_O [68].

**Figure 4:**
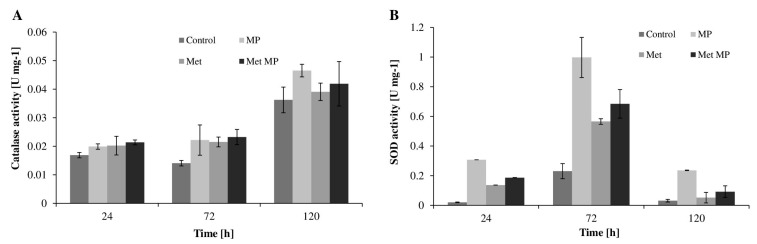
CAT (**A**) and SOD (**B**) activity in *T. harzianum* cells after 24, 72, and 120 h of cultivation with MPs (2 g L^−1^), MET (50 mg L^−1^), MPs and MET mixture, or without the tested pollutants (control). MP—culture with microplastics; MET—culture with metolachlor; MET MP—culture with both metolachlor and microplastics.

As seen in Figure 4, the activity level of both CAT and SOD was increased due to MPs added either separately or in combination with MET. It was observed that on the first day of cultivation in the presence of MPs, the activity of SOD was significantly increased, while there was only a slight change in the level of CAT. In cells derived from 24 h cultures grown in the presence of MPs, MET, and both, the SOD level was 0.30, 0.13, and 0.18 U mg^−1^, respectively, which was 15, 7.5, and 9 times higher, respectively, compared to cells from control cultures (no contaminant added). On the other hand, in the case of CAT, the greatest differences in the level of activity were noted in cells from 72 h cultures containing MPs, and the enzyme activity in these systems was about 40% higher than in control cultures.

#### 2.3.3. Lipid Peroxidation

Overproduction of ROS results in excessive oxidation of membrane lipids. Thus, measuring the level of thiobarbituric-acid-reactive substances (TBARS) is one of the methods to assess the degree of lipid peroxidation.

As presented in Table 4, there were not significant changes in the TBARS level due to the presence of MPs in the 24 h cultures. However, after 72 h of cultivation, the TBARS level in cells from cultures incubated with MPs, MET, or both, was 19.08, 18.21, and 18.24 µM g^−1^, respectively, while it was 14.40 µM g^−1^ in the control cultures. These results confirmed that excess ROS were not fully mitigated by the antioxidative system, resulting in increased lipid peroxidation in MP-treated *T. harzianum* cells. Yu et al. described that *Bacopa* cells exposed to MPs showed increased content of malondialdehyde (MDA), a toxic end product that overaccumulates during lipid peroxidation [69]. Similarly, in a study by Boughattas et al., the cells of earthworm *Eisenia andrei* treated with MPs, 2,4-D, or their mixture showed a significant increase in MDA content [70]. Furthermore, lipid peroxidation was noted in the brain of *D. labrax* fish as a response to the presence of MP as well as as a result of exposure to a mixture of MP and mercury [63]. However, there is still a lack of information on MP-induced oxidative damage in microorganism cells.

To summarize, the possible scenario of events that occur in *T. harzianum* cells in response to the presence of MPs in the growth environment can be outlined as follows. First, MPs cause an increase in the permeability of cell membranes and initiate the intracellular overproduction of ROS. Next, fungal cells counteract the overproduction of ROS by increasing the production of intracellular antioxidants, such as ROS-scavenging enzymes (CAT and SOD). However, this reaction seems to be insufficient. Because of an imbalance between the ROS production and antioxidant capacity, lipid oxidative damage occurs. Similar mechanisms of response to the action of MPs have been characterized in different species of bivalves and summarized in a review by Li et al. [71].

## 3. Materials and Methods

### 3.1. Chemicals

MET, butylated hydroxytoluene, thiobarbituric acid, and 2′,7′-dichlorodihydrofluorescein diacetate (H_2_DCFDA) were purchased from Merck (Germany). Phospholipid standards were purchased from Avanti Polar Lipids (Alabaster, AL, USA). Ethanol, methanol, chloroform, acetonitrile, QuEChERS extraction method ingredients, lipids extraction ingredients, and high-purity solvents used during sample preparation for liquid chromatography–tandem mass spectrometry (LC–MS/MS) analyses were purchased from Avantor (Gliwice, Poland) (>98% purity). All reagents were of analytical grade. Buffers and solutions were prepared in ultrapure water. Stock solutions of MET were prepared with 25 mg mL^−1^ of ethanol.

### 3.2. MP Preparation

LDPE powder (melt index 190 °C/2.16 kg, grain size 100–500 µm) was purchased from Abifor AG (Zürich, Switzerland). The powder was suspended in ethanol (96%) for 2 h for sterilization. Following this, ultrapure water was added to the suspension to achieve an MP concentration of 100 mg mL^−1^. The suspension thus prepared was vortexed and added to the growth medium to obtain 2 g L^−1^ of MPs.

### 3.3. Microorganisms and Growth Conditions

The study was performed on filamentous fungus *T. harzianum* KKP 534 from the Collection of the Industrial Microorganisms of the Institute of Agricultural and Food Industry (IAFB) (Warsaw, Poland), a member of the World Data Centre for Microorganisms (WDCM 212). The spores from 7-day-old cultures on ZT slants containing (g L^−1^): glucose (4.0); Difco yeast extract (4.0); agar (25.0) and malt extract (6° Balling [BLG] up to 1 L) were used for inoculum preparation [72]. The initial preculture was cultivated on a rotary shaker Ecotron (Infors HT) with 160 rpm at 28 °C for 24 h, and then transferred to a fresh medium in a ratio of 1:1 (*v*/*v*) and incubated for the next 24 h. The homogenous preculture (2 mL) was introduced into a 100-mL Erlenmeyer flask containing 18 mL of mineral medium composed of the following components (g L^−1^): K_2_HPO_4_ (4.36), KH_2_PO_4_ (1.7), NH_4_Cl (2.1), MgSO_4_·7H2O (0.2), MnSO_4_ (0.05), FeSO_4_·7H_2_O (0.01), CaCl_2_·2H_2_O (0.03). The medium additionally contained 2% glucose and 1% yeast extract and, if necessery, it was supplemented with MET (at a concentration of 50 mg L^−1^), LDPE microparticles (2 g L^−1^), or both pollutants. Simultaneously, biotic and abiotic controls were also prepared. All cultures were grown for 1–7 days on a rotary shaker at 28 °C.

### 3.4. Dry Weight Mass Analysis

The *T. harzianum* mycelium was separated from the extracellular fluid via filtration with a vacuum pump using previously weighed Whatman filter papers No. 1 (Merck KGaA, Darmstadt, Germany). Then, the mycelium was dried at 100 °C to reach a constant weight. Fungal dry weight was expressed as g L^−1^.

### 3.5. Analytical Methods

#### 3.5.1. Extraction of MET and LC–MS/MS Analysis

MET was extracted from fungal cultures by applying the Quechers method with some modifications [48]. Briefly, the whole fungal cultures were transferred to 50 mL Falcon tubes and homogenized with glass beads (1 mm diameter), acetonitrile (10 mL), and a salt mixture (2 g of MgSO_4_, 0.5 g of NaCl, 0.5 g of C_6_H_5_NaO_7_·2H_2_O, and 0.25 g of C_6_H_6_Na_2_O_7_·1.5H_2_O) using a ball mill Ball Mill MM 400 (Retsch, Haan, Germany). Homogenization was carried out twice for 4 min at a frequency of 30 Hz. Then, the samples were centrifuged for 5 min at 4 °C and 2000× *g* using MPW 260RH centrifuge (MPW Med. Instruments, Warsaw, Poland), and 2 mL of the top layer was collected for further analysis. The samples were diluted in a mixture of methanol and water and transferred to chromatography bottles.

LC–MS/MS analyses were conducted using an Agilent 1200 HPLC system and a QTRAP 3200 mass spectrometer equipped with an electrospray ionization source (ESI), as described by Nykiel-Szymańska et al. [73]. Chromatographic separation was carried out on a Kinetex C18 column (50 mm × 2.1 mm, 5 μm) from Phenomenex (Torrance, CA, USA). The column oven temperature was set at 35 °C. The mobile phase used for separation consisted of water (A) and methanol (B) supplemented with 5 mM ammonium formate. The gradient program was as follows: the solvent gradient was initiated at 20% B and, after 1 min, increased to 90% B for 1 min; then, it was maintained at 90% B for 1.5 min before returning to the initial solvent composition over 1.5 min.

The injection volume was set to 10 µL and the flow rate to 500 µL min^−1^. Pesticide detection was performed using an MS/MS detector working in the multiple reaction monitoring (MRM) positive ionization mode. The monitored MRM pairs were *m*/*z* 284.2–252.2 and *m*/*z* 284.2–176.3 at a retention time of 3.03 min.

#### 3.5.2. Glucose Consumption Analysis

Glucose concentration in the supernatant was determined using an Agilent 1200 HPLC system coupled with a QTRAP 3200 mass spectrometer by performing a flow injection analysis as described by Bernat et al. [45].

#### 3.5.3. Phospholipids Extraction and Analysis

Phospholipids were extracted from *T. harzianum* biomass using a method described previously by Bernat et al. with some modifications [52]. Briefly, 100 mg of fungal biomass washed with distilled water was transferred to a 2 mL Eppendorf tube containing glass beads, 0.66 mL methanol, and 0.33 mL chloroform. The biomass was homogenized for 1 min at a frequency of 30 Hz using a ball mill Ball Mill MM 400 (Retsch, Haan, Germany). The resulting mixture was transferred to another Eppendorf tube, and 0.4 mL of ultrapure water was added. Then, the sample was vortexed and centrifuged at 2000× *g* for 5 min. The organic lower layer was collected, evaporated overnight, and stored at −20 °C for further analysis. The obtained lipid extract was fractionated using an Exion LC AC UHPLC system (Sciex, Framingham, MA, USA). For this, 10 μL of the extract was injected into a Kinetex C18 column (50 mm × 2.1 mm, particle size: 5 μm; Phenomenex (Torrance, CA, USA) at a flow rate of 500 μL min^−1^. The column temperature was maintained at 40 °C. The mobile phases used for fractionation were water (A) and methanol (B), both of which consisted of 5 mM ammonium formate. The gradient program was as follows: the solvent elution was initiated from 70% B and then increased to 95% B over 1.25 min and maintained at 95% B for 6 min before returning to the initial solvent composition over 3 min. The mass spectrometric analysis was carried out using a 4500 Q-TRAP mass spectrometer (Sciex, Framingham, MA, USA) equipped with an ESI source under the following conditions: spray voltage −4500 V, curtain gas 25 psi, nebulizer gas 50 psi, turbo gas 60 psi, and ion source temperature 600 °C. Data analysis was performed using the Analyst™ v1.6.3 software (Sciex, Framingham, MA, USA). Qualitative and quantitative analyses were carried out as described in our previous papers [47,52].

#### 3.5.4. Fatty Acids Analysis

For fatty acids analysis, 0.75 mL of the obtained lipid extract, prepared according to the steps described above, was transferred to a screw-capped glass test tube, and then 0.1 mL toluene and 0.15 mL HCl solution (8.0%) were added. The tube was vortexed and incubated overnight at 45 °C. After cooling to room temperature, 0.5 mL hexane and 0.5 mL water (deionized) were added to the extract of fatty acid methyl esters (FAMEs). The tube was vortexed, and then 0.2 mL of the hexane layer was moved to the chromatographic vial, and 1.6 µL of the extracted samples was analyzed.

The FAME analysis was carried out with an Agilent Model 7890 gas chromatograph equipped with a 5975C mass detector. A capillary column HP 5 MS methyl polysiloxane (30 m × 0.25 mm i.d. × 0.25 mm ft) was used with helium as a carrier gas. The temperature of the column was maintained at 60 °C for 3 min and was increased to 212 °C at a rate of 6 °C min^−1^, then to 245 °C at a rate of 2 °C min^−1^, and finally to 280 °C at a rate of 20 °C min^−1^, at which it was held for 10 min. Split injection of the injection port was performed at 250 °C. Fungal fatty acids were identified through comparison with authenticated reference standards from Sigma–Aldrich (Supelco, Bellefonte, PA, USA), and the results were expressed as a percentage of the total amount of fatty acids.

#### 3.5.5. Lipid Peroxidation Assay

The degree of lipid peroxidation was measured spectrophotometrically (FLUOstar microplate reader, BMG Labtech with Omega version 5.10 R2 software) as the content of TBARS, as described by Jo and Ahn [74] with modifications made previously by our team [52]. The level of lipid peroxidation was expressed as micromolar of TBARS calculated per gram of wet biomass.

#### 3.5.6. Membrane Permeability

The membrane permeability was assessed using propidium iodide, as described previously by Jasińska et al. [75]. The results were expressed as fluorescence units per milligram of the dry fungal biomass.

#### 3.5.7. Enzyme Activity Determination

The washed fresh mycelium (500 mg) was homogenized in an ice-cold mortar with 5 mL of 50 mM sodium phosphate buffer (pH 7) containing 1% polyvinylpyrrolidone, 10 mM sodium ascorbate, and 1 mM ethylene diamine tetraacetic acid. The mixture was collected in 15 mL Falcon tubes and centrifuged at 10,000× *g* at 4 °C for 15 min. The obtained supernatant was transferred to new tubes and kept on ice. The activity of SOD and CAT was measured as previously described in detail by our group [36]. CAT activity was assessed spectrophotometrically by measuring H_2_O_2_ degradation at 240 nm, while SOD activity was determined spectrophotometrically by the reduction of nitrotetrazolium blue chloride at 540 nm. The values of CAT and SOD activity were expressed as units per milligram of protein. The protein content in the tested samples was assayed using the Bradford method.

#### 3.5.8. Measurement of Intracellular Peroxynitrate Anion/Hydroxyl Radical Anion

The total intracellular levels of peroxynitrate anion/hydroxyl radical anion (HO•/ONOO−) in the fungal biomass were determined with the cell-permeant H_2_DCFDA (Sigma Aldrich, St. Louis, MO, USA) assay as described by Liu et al. [76] and modified by Nykiel-Szymańska [36]. The results were expressed as a percentage of the green fluorescence area compared to the total hyphal area.

### 3.6. Statistical Analysis

The experiment was carried out in three replicates, and the standard deviation was determined. One-way analysis of variance was performed using Microsoft^®^ Excel^®^ (Microsoft Corporation, Washington, DC, USA). Differences at *p* < 0.05 were considered significant. Excel 2013 was used to present averaged quantitative data in the form of a heat map.

## 4. Conclusions

It can be concluded that the biomass production and MET biodegradation by the fungus *T. harzianum* is not affected by MPs. However, MPs strongly influenced the lipid profile of *T. harzianum* by modifying fatty acid content and classes of lipids. The addition of MPs to the culture resulted in the reduction of PC content and an increase in PE content. As a result, the PC/PE ratio, which provides information about the integrity of the cell membrane, decreased. Moreover, the degree of unsaturation of the fatty acids increased in the systems containing MPs. This led to a reduction in the integrity of the cell membrane, which was confirmed in a permeation assay with propidium iodide. As a result, induced generation of ROS, increased lipid peroxidation, and activity of antioxidant enzymes (CAT and SOD) were observed. All these results suggest that MPs generate oxidative stress in *T. harzianum* cells. To our knowledge, this is the first report indicating the induction of oxidative stress in fungal cells under the influence of plastic microparticles. The results presented in the study additionally indicate that despite unfavorable conditions, *T. harzianum* can effectively biodegrade MET in the presence of MP. Microplastic and pesticides co-pollute the soil and water environment. Therefore, testing the resistance of fungi to multicomponent pollutants and the ability to eliminate them is a very urgent issue. *Trichoderma* fungi are an important factor in biological plant protection against both biotic and abiotic stressors. The presented work may help in understanding this process and is also a starting point for further research, including the treatment of soil contaminated with pesticides (including MET) and MPs with the strain *T. harzianum*.

## Figures and Tables

**Table 1:** Phospholipids composition and PC/PE and PI/PS ratios determined in the *T. harzianum* cells after 24, 48, 72, 96, and 120 h of cultivation with MPs (2 g L^−1^), MET (50 mg L^−1^), MPs and MET mixture, or without the tested pollutants.

Time of Cultivation [h]	Culture	Relative Abundance [%]						
		PA	PC	PE	PI	PS	PC/PE	PI/PS
24	Control	1.89	44.32	48.33	4.37	1.10	0.92	3.99
	MP	2.52	27.24	62.46	6.53	1.24	0.44	5.25
	MET	2.21	43.48	47.31	5.49	1.51	0.92	3.64
	MET MP	1.39	29.46	63.18	4.89	1.08	0.47	4.51
48	Control	1.48	32.27	61.17	3.63	1.46	0.53	2.49
	MP	2.29	15.90	70.47	8.75	2.59	0.23	3.38
	MET	1.95	19.17	69.70	7.22	1.96	0.28	3.69
	MET MP	0.67	15.50	73.84	8.08	1.91	0.21	4.22
72	Control	0.34	30.49	64.84	2.82	1.51	0.47	1.86
	MP	0.32	29.90	65.55	2.92	1.31	0.46	2.23
	MET	0.26	25.64	68.15	4.21	1.73	0.38	2.43
	MET MP	1.33	26.69	67.80	3.18	1.00	0.39	3.19
96	Control	0.13	48.41	48.08	2.75	0.63	1.01	4.37
	MP	0.44	42.45	52.63	3.58	0.89	0.81	4.00
	MET	0.09	62.56	34.16	2.74	0.45	1.83	6.10
	MET MP	0.27	32.64	63.03	3.06	1.00	0.52	3.07
120	Control	0.25	64.38	33.14	1.84	0.40	1.94	4.61
	MP	0.12	61.08	34.87	3.42	0.51	1.75	6.70
	MET	0.05	63.66	32.90	3.05	0.34	1.93	8.98
	MET MP	0.04	66.44	29.85	3.16	0.50	2.23	6.38

MP—culture with microplastics; MET—culture with metolachlor; MET MP—culture with both metolachlor and microplastics; PA—phosphatidic acid; PC—phosphatidylcholine; PE—phosphatidylethanolamine; PI—phosphatidylinositol; PS—phosphatidylserine.

**Table 2:** Influence of MPs (2 g L^−1^), MET (50 mg L^−1^), MPs, and MET mixture on the fatty acid profile of *T. harzianum*.

Culture	Relative Abundance [%]				Unsaturation Index
	16:0	18:0	18:1	18:2	
Control	37.70	17.59	22.82	21.89	66.61
MP	32.17	14.36	26.59	26.89	80.06
MET	31.88	11.70	27.16	29.25	83.57
MET MP	33.51	15.04	24.02	27.44	75.47

MP—culture with microplastics; MET—culture with metolachlor; MET MP—culture with both metolachlor and microplastics.

**Table 3:** Effect of MPs (2 g L^−1^) and/or MET (50 mg L^−1^) on *T. harzianum* membrane permeability (expressed as propidium iodide fluorescence in fungal biomass) after 24, 72, and 120 h of cultivation.

Culture	Membrane Permeability Expressed as Propidium Iodide Fluorescence Intensity (U·mg^−1^ of Dry Biomass)		
	24 h	72 h	120 h
Control	18,231.10 ± 8805.60	56,703.85 ± 2806.94	51,053.15 ± 1510.17
MP	51,847.00 ± 1179.00	33,750.92 ± 5951.27	30,214.06 ± 1020.57
MET	45,799.75 ± 7376.89	44,132.70 ± 1931.15	29,158.80 ± 868.97
MET MP	20,119.80 ± 2027.42	29,740.88 ± 470.86	27,784.66 ± 7612.87

MP—culture with microplastics; MET—culture with metolachlor; MET MP—culture with both metolachlor and microplastics.

**Table 4:** Effect of MPs (2 g L^−1^) and/or MET (50 mg L^−1^) on *T. harzianum* lipid peroxidation (expressed as TBARS level) after 24, 72, and 120 h of cultivation.

Culture	TBARS Level [µM g^−1^ of Wet Biomass]		
	24 h	72 h	120 h
Control	7.83 ± 1.63	14.40 ± 1.37	15.79 ± 0.31
MP	7.83 ± 0.80	19.08 ± 1.91	18.47 ± 2.70
MET	7.53 ± 0.26	18.21 ± 0.92	18.37 ± 0.83
MET MP	7.67 ± 0.77	18.24 ± 1.55	22.01 ± 0.46

MP—culture with microplastics; MET—culture with metolachlor; MET MP—culture with both metolachlor and microplastics.

## Data Availability

The data presented in this study are available on request from the corresponding author.

## Supplementary Materials

The following supporting information can be downloaded at: https://www.mdpi.com/article/10.3390/ijms232112978/s1.
