# Supplementary material for: Microplastic-Induced Oxidative Stress in Metolachlor-Degrading Filamentous Fungus Trichoderma harzianum
Source: Int J Mol Sci. 2022 Oct 26;23(21):12978. doi: 10.3390/ijms232112978 (PMC9658726; doi:10.3390/ijms232112978)
Supplement: Supplementary file 1 [file ijms-23-12978-s001.zip › ijms-1971004-supplementary.pdf]

Table S1. Content of the molecular species of phospholipids determined in the *T. harzianum* cells after 24, 48, 72, 96, and 120 hours of cultivation with MPs (2 g L^{-1}), MET (50 mg L^{-1}), MPs, and MET mixture, or without the tested pollutants (control).

	Time [h]																			
**Phospholipid species**	**24**				**48**				**72**				**96**				**120**			
	**C**	**MP**	**MET**	**MIX**	**C**	**MP**	**MET**	**MIX**	**C**	**MP**	**MET**	**MIX**	**C**	**MP**	**MET**	**MIX**	**C**	**MP**	**MET**	**MIX**
**PA 16;0 18;2**	1,89	2,52	2,21	1,39	1,48	2,29	1,95	0,67	0,34	0,32	0,26	1,33	0,13	0,44	0,09	0,27	0,25	0,12	0,05	0,04
**PC 16;0 18;2**	4,74	2,14	2,93	1,27	1,80	1,49	1,35	0,61	2,55	1,79	1,83	1,31	3,32	1,88	1,93	1,96	2,70	2,09	2,74	1,82
**PC 16;0 18;1**	2,20	1,74	0,81	0,62	0,88	0,59	0,25	0,27	0,93	0,67	0,51	0,62	1,40	0,73	0,21	0,67	0,89	0,28	0,43	0,25
**PC 18;2 18;2**	23,27	14,21	25,73	17,10	20,61	8,48	12,48	10,19	19,08	21,31	16,59	17,86	32,13	30,76	50,18	21,33	52,22	49,40	50,69	54,95
**PC 18;2 18;1**	9,41	6,38	9,80	6,75	6,56	2,97	3,87	3,72	6,21	4,44	4,92	5,24	9,03	6,69	8,30	6,36	7,34	8,00	8,58	8,37
**PC 18;1 18;1**	3,31	2,40	3,80	3,41	2,01	1,74	1,16	0,66	1,62	1,62	1,72	1,62	2,21	2,27	1,91	2,22	1,13	1,25	1,19	1,04
**PC 18;0 18;1**	1,38	0,38	0,42	0,30	0,40	0,62	0,05	0,05	0,09	0,07	0,07	0,04	0,32	0,13	0,03	0,09	0,10	0,05	0,03	0,02
**PE 16;0 18;2**	29,92	40,14	29,36	36,54	38,24	40,65	43,83	44,36	41,27	39,30	42,86	44,87	31,76	32,23	18,94	39,70	20,88	20,69	20,21	16,89
**PE 16;0 18;1**	4,83	5,41	6,15	6,66	8,49	9,91	7,93	9,02	8,31	7,72	8,46	6,57	4,46	4,55	2,17	6,60	1,89	2,10	1,29	1,43
**PE 18;2 18;2**	7,53	8,82	6,17	10,90	7,45	8,73	8,85	9,84	8,11	10,07	8,32	9,33	6,38	8,84	9,08	8,73	7,91	7,73	8,43	8,99
**PE 18;2 18;1**	2,00	2,96	2,50	3,77	3,08	4,85	4,26	4,76	3,28	4,31	4,77	3,55	2,41	3,43	2,63	3,84	1,44	2,13	1,84	1,70
**PE 18;1 18;1**	1,62	2,80	1,80	3,55	1,72	3,48	2,17	4,83	2,30	2,68	2,38	2,51	1,53	2,42	0,89	2,69	0,59	1,14	0,49	0,60
**PE 18;2 18;0**	1,96	1,99	1,07	1,53	1,70	2,26	2,46	0,84	1,31	1,29	1,27	0,82	1,42	1,11	0,43	1,42	0,36	1,04	0,63	0,22
**PE18;1 18;0**	0,47	0,33	0,26	0,23	0,48	0,58	0,18	0,19	0,25	0,19	0,10	0,15	0,11	0,06	0,01	0,06	0,07	0,04	0,01	0,02
**PI 16;0 18;2**	1,72	2,63	2,36	2,38	1,41	4,46	3,39	3,81	1,07	1,56	1,66	1,31	1,32	1,67	1,74	1,31	1,17	1,87	2,04	2,07
**PI 16;0 18;1**	1,16	1,41	1,47	1,02	0,86	2,26	2,03	1,90	0,80	0,67	1,17	0,55	0,68	1,07	0,48	0,69	0,33	0,64	0,45	0,48
**PI 18;2 18;2**	0,23	0,60	0,33	0,29	0,20	0,30	0,47	0,46	0,14	0,11	0,20	0,18	0,14	0,21	0,11	0,13	0,13	0,13	0,26	0,19
**PI 18;1 18;2**	0,74	0,94	0,54	0,67	0,64	1,15	0,48	0,78	0,36	0,29	0,71	0,47	0,35	0,41	0,09	0,43	0,13	0,31	0,13	0,23
**PI 18;1 18;1**	0,25	0,38	0,34	0,17	0,22	0,21	0,46	0,47	0,17	0,13	0,14	0,22	0,12	0,10	0,05	0,16	0,01	0,06	0,01	0,04
**PI 18;0 18;2**	0,14	0,45	0,29	0,23	0,19	0,21	0,25	0,38	0,19	0,12	0,21	0,33	0,10	0,09	0,26	0,29	0,06	0,39	0,16	0,15
**PI 18;0 18;1**	0,12	0,13	0,16	0,14	0,12	0,17	0,13	0,27	0,09	0,05	0,12	0,12	0,03	0,03	0,01	0,05	0,01	0,02	0,00	0,00
**PS 16;0 18;2**	0,23	0,52	0,45	0,15	0,58	0,55	0,61	0,57	0,46	0,29	0,34	0,25	0,18	0,30	0,13	0,24	0,10	0,10	0,06	0,17
**PS 16;0 18;1**	0,38	0,50	0,71	0,52	0,65	1,51	1,03	0,98	0,58	0,63	0,67	0,45	0,25	0,33	0,11	0,30	0,12	0,10	0,05	0,13
**PS 18;2 18;2**	0,49	0,23	0,35	0,41	0,23	0,53	0,31	0,36	0,48	0,38	0,72	0,30	0,20	0,26	0,20	0,45	0,18	0,30	0,23	0,20
